# Randomized Controlled Trial of Simulation vs Standard Training for Teaching Medical Students High-quality Cardiopulmonary Resuscitation: The Methodological Issue

**DOI:** 10.5811/westjem.2019.8.43700

**Published:** 2019-10-14

**Authors:** Reza Farahmand Rad, Akram Zolfaghari Sadrabad, Shahab Rezaeian

**Affiliations:** Clinical Research Development Center, Imam Reza Hospital, Kermanshah University of Medical Sciences, Kermanshah, Iran

To the Editor:

We read with great interest the paper by McCoy et al.^1^ published in the January 2019 issue of your valuable journal. The authors sought to evaluate the comparative effectiveness of high-fidelity simulation training vs standard manikin training for teaching medical students, using the American Heart Association guidelines for high-quality cardiopulmonary resuscitation (CPR). They concluded that high-fidelity simulation training was better than low-fidelity CPR manikin training.

Although this study was done in detail with an interesting result, there are some important methodological issues which should be considered to improve its application in practice and for future research:

1. The participants wereall fourth-year medical students, but there was no information about baseline data regarding their characteristics. Wouldn’t this issue be important as to whether or not the characteristics between the two groups were comparable?
2. There were two comparative groups in the study, but the authors used Kruskal-Wallis rank sum test without any adjustment. Mann-Whitney U test is commonly used to compare two sample means when the distribution is non-normal.^2^
3. It would have been better to refer to a related reference for calculating sample size in the study. Based on what justification was an effect size of five millimeters considered for comparing two groups?
4. We believe that some confounding variables such as previous experience, education, or interest in their own field may have had an effect on the results.^3^
5. The method of data collection appears to be missing or was not made clear to the reader.
6. Real-time feedback can increase the average of physical and mental workloads, and the quality of CPR then improves^4^ with higher reported physical workloads. In this study, it would have been better to do the training in the two groups by the mentioned method, and the two groups could then have been evaluated after a time interval.
7. We thank the authors for reporting the limitations of their study honestly. In one limitation, the authors declare that increasing the number of outcome measures increases the potential for a type I error. In this analysis a two-group comparison was done, not multiple comparisons.^5^
